# Reliability of species detection in 16S microbiome analysis: Comparison of five widely used pipelines and recommendations for a more standardized approach

**DOI:** 10.1371/journal.pone.0280870

**Published:** 2023-02-16

**Authors:** Andreas Hiergeist, Jean Ruelle, Stefan Emler, André Gessner

**Affiliations:** 1 Institute of Clinical Microbiology and Hygiene, University Hospital Regensburg, Regensburg, Germany; 2 SmartGene Services SARL, Lausanne, Switzerland; University of Nebraska-Lincoln, UNITED STATES

## Abstract

The use of NGS-based testing of the bacterial microbiota is often impeded by inconsistent or non-reproducible results, especially when applying different analysis pipelines and reference databases. We investigated five frequently used software packages by submitting the same monobacterial datasets to them, representing the V1-2 and the V3-4 regions of the 16S-rRNA gene of 26 well characterized strains, which were sequenced by the Ion Torrent™ GeneStudio S5 system. The results obtained were divergent and calculations of relative abundance did not yield the expected 100%. We investigated these inconsistencies and were able to attribute them to failures either of the pipelines themselves or of the reference databases they rely on. On the basis of these findings, we recommend certain standards which should help to render microbiome testing more consistent and reproducible, and thus useful in clinical practice.

## Introduction

Microbiome sequencing enables new insights into the role of microorganisms in various pathologies, as well as into their roles when interacting with the host immune system [1]. High-throughput sequencing techniques could enable broad-range molecular diagnostics, not only from primary sterile material like cerebrospinal fluid, organ tissue or vitreous aspirates, but also for the detection of pathogens within complex communities of commensal microorganisms. The transition of microbiome analysis into routine diagnostics with clinical application is still hampered by the lack of standardization, that renders the reproducibility and comparison of such results difficult [2]. Methodological variations during all steps from sampling, through to wet-lab processes, including cell lysis, PCR amplification, library preparation and high-throughput sequencing platforms, have been extensively analyzed in various studies [3–5]. The choice of target region to be sequenced as well as the analysis software and databases used also have an impact on the results and thus need to be evaluated and understood. Our study therefore focuses on this aspect of the workflow.

A prerequisite for implementation of microbiome sequencing in clinical diagnostics is the ability to accurately determine the presence or absence of pathogenic and beneficial species, and their abundance. Reliable species-level identification and quantification is necessary to identify compositional shifts over time within complex sample matrices. These requirements hold true also for pre-clinical and clinical studies which build the basis for valid scientific conclusions and interpretation for certain pathologies.

The gene (named here 16S) which encodes for the small ribosomal subunit, 16S rRNA, is the most widely used phylogenetic marker and sequences for all recognized species are available. The 16S gene has 9 variable regions, but not all have the same potential to differentiate species [6]. Since NGS sequencing often relies on the generation of rather short reads, many authors focus on the V3-V4 region, thus often restricting their analysis to the genus-level because species differentiation cannot be achieved. Other variable regions such as the V1-V2 region hold the promise for better species differentiation [7], but would need to be evaluated for the purpose of microbiome analysis. Recently, sequencing of full-length 16S became available and Johnson *et al*. demonstrated differentiation of intra-genomic 16S polymorphisms within one genome for strain-level discrimination [6]. However, this technology is still rather complex, expensive and sensitivity is an issue; therefore, routine applications focus rather on partial 16S analyses. In our study we compare results obtained with the V1-V2 and V3-V4 regions which are easily covered by widely used sequencing technologies.

After sequencing is performed, reads need to be matched against a reference database to assign a species. Frequently used clustering or binning algorithms group reads or contigs into operational taxonomic units (OTU) based on a predefined similarity cutoff, usually e.g. 97%; consensus sequences are then used for mapping the most similar reference sequence. Given the high resemblance of 16S sequences of some close species, other approaches favor comparing all divergent contigs against the references, without prior clustering. In our study we have evaluated both types of approaches.

Currently, multiple 16S reference databases exist, including publicly available ones such as SILVA [8], Greengenes [9], All Species Living Tree Project (LTP, [10]), Genome Taxonomy Database (GTDB, [11]), as well as commercial products such ase EzTaxon (ezbiocloud.com, [12]), or SmartGene (smartgene.com, Switzerland). All these databases differ significantly regarding their content (partial sequences vs full genomes vs type strains etc), their size, how they are curated, and how frequently they are updated. Obviously, the choice of database impacts on the results one can obtain and therefore we included several databases in our evaluation.

Given the difficulties often encountered with non- comparable or non-reproducible results, our study explores the impact of the following choices: target variable region of the 16S, the pipeline (matching algorithms against references) and the databases (content, representation of the Kingdom). We used a very simplified microbiome model based on 26 well characterized and clinically relevant bacterial species. Ideally, all results should yield a fraction of 100% of sequences assigned correctly for the species under investigation; in cases where this was not achieved, we analyzed in more depth the underlying causes and provide some recommendations.

## Material and methods

### Cultivation and sources of bacterial strains

Bacterial isolates used in this study were obtained or isolated from different sources. Each sample represents a single strain originated from pure cultures. These strains have been selected primarily to cover a broad spectrum of the bacterial kingdom. Species from all bacterial phyla that can be frequently found as part of the human microbiota (Actinobacteria, Bacteroidetes, Firmicutes, Proteobacteria) have been included. The majority of strains were either obtained from the DSMZ culture collection (www.dsmz.de, Braunschweig, Germany), or from quality control material used in international external quality assessment schemes (EQAS). These were supplemented by strains which have been isolated from patient specimens during routine microbiological diagnostics or from cecal content of C75BL/6 wildtype mice. Initial identification of species was conducted according to DIN EN ISO 15189 accredited methods including MALDI-Tof mass spectrometry using the MALDI Biotyper® system (Bruker Daltonics Inc., Billerica, MA, USA). The identified species names and all sources and conditions used for cultivation of these strains are summarized in Table 1.

**Table 1:** Overview on bacterial strains and isolates used in this study and the culture media used.

Strain Number	Species	Source	Cultivation
1	*Agrobacterium radiobacter*	DSM 30147	Aerobic, Luria-Bertani (LB) agar
2	*Alcanivorax borkumensis*	DSM 11573	Aerobic, Marine Broth (Difco)
3	*Alicyclobacillus acidiphilus*	DSM 14558	Aerobic, Alicyclobacillus medium 402 (DSMZ)
4	*Bacillus licheniformis*	Clinical isolate	Aerobic, Columbia blood agar (Oxoid)
5	*Bacteroides caccae*	Clinical isolate	Anaerobic, Schaedler-KV agar
6	*Bacteroides fragilis*	ATCC 25285	Anaerobic, Schaedler -KV agar
7	*Bacteroides thetaiotaomicron*	ATCC 29741	Anaerobic, Schaedler-KV agar
8	*Bifidobacterium longum*	Clinical isolate	Anaerobic, Bifidobacterium agar, modified (BD)
9	*Butyricimonas virosa*	Blood culture isolate	Anaerobic, Schaedler Anaerobe agar (Oxoid)
10	*Clostridium tertium*	INSTAND bacteriology ring trial round 2006	Anaerobic, Schaedler Anaerobe agar (Oxoid)
11	*Enterococcus durans*	INSTAND bacteriology ring trial round 2000	Aerobic, Culumbia blood agar (Oxoid)
12	*Enterococcus faecium*	ATCC 6057	Aerobic, Culumbia blood agar (Oxoid)
13	*Enterococcus gallinarum*	Clinical isolate	Aerobic, Culumbia blood agar (Oxoid)
14	*Escherichia coli*	DSM 6897	Aerobic, Luria-Bertani (LB) agar
15	*Salinibacter ruber*	DSM 13855	Aerobic, Salinibacter medium 936 (DSMZ)
16	*Staphylococcus aureus*	ATCC 29213	Aerobic, Culumbia blood agar (Oxoid)
17	*Idiomarina loihiensis*	DSM 15497	Aerobic, Marine Broth (Difco)
18	*Lacticaseibacillus rhamnosus*	Murine isolate	Anaerobic, Lactobacilli MRS agar (Difco)
19	*Lactobacillus gasseri*	Clinical isolate	Anaerobic, Lactobacilli MRS agar (Difco)
20	*Lactococcus lactis*	Murine isolate	Anaaerobic, Schaedler Anaerobe Agar (Oxoid)
21	*Leuconostoc mesenteroides*	Clinical isolate	Aerobic, Culumbia blood agar (Oxoid)
22	*Ligilactobacillus salivarius*	Murine isolate	Lactobacilli MRS agar (Difco)
23	*Limosilactobacillus reuteri*	DSM 12246	Lactobacilli MRS agar (Difco)
24	*Paenibacillus aceti*	Clinical isolate	Aerobic, Culumbia blood agar (Oxoid)
25	*Paenibacillus barengoltzii*	Clinical isolate	Aerobic, Culumbia blood agar (Oxoid)
26	*Streptococcus oralis*	Clinical isolate	Aerobic, Culumbia blood agar (Oxoid)

Bacterial species were cultivated from cryobank stocks (Mast Diagnostica, Reinfeld, Germany) stored at—80°C. One bead containing cryopreserved bacterial cells was transferred to agar plates and further streaked out using 10 μl inoculation loops. Growth media are summarized in Table 1. *Lactobacillus* and *Rhizobium* species were cultivated at 30°C, all other species at 37°C. Anaerobic bacteria were cultivated under oxygen-free atmosphere (85% N2, 10% CO2, 5% H2) in an anaerobic jar using the Advanced® Anoxomat™ system (Augusta Laborbedarf GmbH). After growth of colonies, cultivation was repeated by transferring a single colony to a new agar plate. Species designation was confirmed by sanger-based sequencing on a ABI PRISM® 310 Genetic Analyzer (Life Technologies) using primers S-D-Bact-0008-a-S-16 and S-D-Bact-1492-a-A-16 [13].

### Extraction of nucleic acids

A total of 3–5 colonies of each bacterial species were suspended into 500 μl of sterile PBS buffer using an inoculation loop and vortexed to obtain homogeneous cell suspensions. Mechanical disruption of bacterial cells was achieved by repeated bead beating by means of a TissueLyser II (Qiagen, Hilden, Germany) for 5 min at 30 Hz using Lysing Matrix Y (0.5 mm diameter yttria-stabilized zirconium oxide beads; MP Biomedicals, Eschwege, Germany). Nucleic acids were purified from crude cell lysates by means of the MagNA Pure 96 instrument (Roche, Mannheim, Germany) using the MagNA Pure 96 DNA and Viral NA Large Volume Kit (Roche). Total amount and purity of total nucleic acids was measured spectrophotometrically using the NanoDrop 1000 instrument (Thermo Fisher Scientific, Waltham, USA).

### Semiconductor-based sequencing of bacterial 16S rRNA genes

Concentrations of nucleic extracts were normalized to 5 nanogram per μL. Subsequently, V1 to V2 and V3 to V4 hyper-variable regions of bacterial 16S rRNA genes were amplified by PCR in two separate reactions from a total of 5 ng DNA using the Platinum II Taq Hot-Start DNA Polymerase (Thermo Fisher Scientific). For amplification of V1 to V2 regions, primer S-D-Bact-0008-c-S-20 containing a 10-bp sample-specific barcode sequence and the IonTorrent-specific sequencing adaptor A together with reverse primer S-D-Bact-0517-a-A-18 containing a 3’-P1 adapter sequence were used. For amplification of V3 to V4 regions, primers S-D-Bact-0341-b-S-17 and S-D-Bact-0785-a-A-21 were used containing the same barcode and adapter sequences. After 30 PCR cycles, amplicons were analyzed by agarose gel electrophoresis. Identical volumes of V1-V2 and V3-V4 PCR reactions were combined for each sample and purified twice using MagSi-NGSprep Plus beads (Steinbrenner, Wiesenbach, Germany) applying a 0.8 beads to DNA ratio. Copy numbers of amplicons containing sequencing-adaptors were determined using the KAPA Library Quantification IonTorrent Kit (Roche) and pooled to equimolar concentrations of each amplicon. A total of 120 attomol of the final library pool was subjected to isothermal amplification using the Ion Chef instrument before running 1350 flow cycles during high-throughput sequencing on an Ion Torrent™ GeneStudio S5 Plus machine (Thermo Fisher Scientific).

### Pre-processing of sequencing reads for further analysis

Signal processing and base calling was performed using the Torrent Suite Software Version 5.12 without additional quality trimming of sequencing reads. Cutadapt 3.1 [14] was used for removal of 5’- and 3’- adapter sequences, for the demultiplexing according to the barcode sequences and the splitting into V1-2 and V3-4 regions after matching the 5’ends with the forward primer sequence. Resulting FASTQ files were then uploaded to the various analysis pipelines for further processing.

### Analysis of preprocessed reads using different analysis pipelines and databases

Preprocessed FASTQ files were subjected to further analysis using five analysis pipelines and reference databases as summarized in Table 2. Divisive Amplicon Denoising Algorithm pipelines (dada2, version 1.16 [15]) were used in combination with SILVA, LTP and GTDB databases, a vsearch pipeline [16] used the Greengenes database, the SmartGene pipeline relied on its proprietary 16S Centroid database. With the exception of the SmartGene pipeline where the database is integrated in a cloud service, database FASTA files were downloaded directly from the respective websites (Table 2).

**Table 2:** Overview of versions and characteristics of analysis pipelines and 16S reference databases used in this study.

Abbreviation	Reference Database /Pipeline	Analysis pipeline	DB Release	DB Release Date	Sequences	Link
d2.GTDB	Genome Taxonomy Database (GTDB)	dada2	05-RS95	17th July 2020	194,600 ^(1)^ (redundant)	https://gtdb.ecogenomic.org/
d2.LTP	All-Species Living Tree Project (LTP)	dada2	LTPs132 SSU	June 2018	13,903 (non-redundant)	https://www.arb-silva.de/projects/living-tree/
d2.SILVA	SILVA	dada2	SSU r138	December 2019	112,585 ^(3)^ (redundant)	http://www2.decipher.codes/Downloads.html
SG	SmartGene 16S Centroid	SmartGene (IDNS -5 v.3.7.0)	2.2.0_16S_r144u034	18th May 2020	15,960 (non-redundant)	https://www.smartgene.com
vs.GG	Greengenes	vsearch	13_5	May 2013	1,262,986 ^(2)^ (redundant)	http://greengenes.secondgenome.com/?prefix=downloads/greengenes_database/gg_13_5/

(1) The Genome Taxonomy Database (GTDB) was constructed by average nucleotide identity and assignment of tentative placeholder species names. Phylogeny based on 120 ubiquitous single-copy proteins was used to propose taxonomic classification.

(2) The total of 1,262,986 near full-length SSU sequences are clustered to 99 percent sequence identity (203,452 sequences). Greengenes uses a NCBI nomenclature based on de novo tree inference and ismaintained and curated by an international consortium of 4 research groups.

(3) The original non redundant (NR) Silva Database release (https://www.arb-silva.de/documentation/release-138/) contains 510,984 bacterial and archaeal 16S rRNA sequences. Here we used a modified SILVA SSU r138 datbase (December 2019) which is provided by the DECIPHER package (http://www2.decipher.codes/Downloads.html). This repository contains taxonomy to the genus-level. Here, putative chimeric sequences, sequences with more than 10 ambiguities, sequences classified to non-basal taxons have been removed resulting into 112,585 database entries.

Prior to the analysis with dada2 and vsearch pipelines, low-quality bases were trimmed using Trimmomatic version 0.39 [17] in a 25 bp sliding window approach, with a phred -score cutoff of 15 and a length cutoff of 150 bases. For dada2 processings, reads with a maximum of five expected errors were denoised applying an OMEGA_A and OMEGA_P cutoff of 1e-30 in conjunction with a homopolymer gap penalty of -1 [15]. Chimeric sequences were detected and removed with the removeBimeraDenovo command; thereafter these reads were then subjected to the IDTAXA algorithm of DECIPHER 2.18.1 [18] for taxonomic classification of all detected amplicon sequence variants (ASV) based on 200 iterations with a bootstrap threshold of 40% as confidence value for assigning a taxon with certainty. For dada2 processing with the SILVA database, the training set provided with DECIPHER was applied, which is annotated to the genus-level only [18]. For the dada2 in combination with the LTP and GTDB databases, new training sets for the IDTAXA classifier were set up from downloaded FASTA files following the DECIPHER tutorial. Relative abundances represent the fraction of reads assigned to the amplicon sequence variants obtained. These reads are summarized at different taxonomic levels.

For data analysis with the SmartGene pipeline, FASTQ files were uploaded to the protected cloud-platform. The **SmartGene** IDNS-5 Bacteria 16S Microbiome App is a commercial CE-IVD labeled automated cloud application service of SmartGene (SmartGene, Unteraegeri, Switzerland, www.smartgene.com). It uses a proprietary SmartGene "16S Centroid" database of non-redundant representative bacterial 16S rRNA sequences covering 15’960 species across 3’161 genera as of May 2020, which is maintained and updated using AI and algorithms (patent #EP02215578). A sliding-window filter eliminates low quality sections of reads and the resulting contigs are mapped against the reference sequences without prior binning. Results are grouped according to match quality (e.g. % of coverage, number of mismatches, etc), match specificity (matching a single species or not), and match consistency (close matches belonging to the same genus). The system produces a confidence score for the matching taxon and if a species cannot be called specifically, the system assigns the next taxon level and indicates all possibly matching species and genera. Results are displayed in a table format, along with the number of reads and the relative abundance, and can be consolidated to the species, genus, and family levels, along with a dynamic Krona diagram. Abundances are measured by the count of reads mapped to a specific species/genus/family.

The vsearch pipeline in combination with the Greengenes database subjected reads with a maximum of 5 expected errors to the downstream analysis: a unoise3 algorithm with minimum size of 2 and an alpha value of 8 was used for denoising, followed by a chimera filtering step using uchime3 [19]. Reads were then pre-sorted by length and clustered at 98.5% sequence identity applying the cluster_fast algorithm prior to mapping them to the Greengenes database using the SINTAX algorithm of vsearch with default parameters [16].

### Comparison of results obtained on the basis of species identification and relative abundances

Relative abundances on the species and genus level were either obtained from the SmartGene pipeline (csv export) or were calculated in R using the phyloseq package version 1.34 [20]. Combined barplots were generated using the ggubr 0.2.4 package. Relative abundances of all analysis pipelines were compared for each sample on the genus and species levels. A relative abundance level of more than 95 percent were defined as acceptable classification result.

### Re-assessment of performance after in-depth analysis of the results

We analyzed the sequence distribution of the bacterial strains within all databases used as well as presence of closely related species. Therefore, full-length 16S centroid sequences of all species used were obtained from the SmartGene IDNS3 module which were considered as reference sequences for the corresponding species. To assess species-level discrimination, the seqs.pcr command of mothur version 1.45 was used to trim all reference sequences to the exact regions covered by the amplification primers for the V1-2 and the V3-4 region respectively [21]. These references represent the ideal situation where all reads cover the full amplicon length. Resulting V1-2 and V3-4 sequences were uploaded to the SmartGene IDNS3 module to search for best matching centroid sequences for all species and both 16S regions. Results tables were sorted by sequence identities and mismatches.

For all other pipelines used, local BLAST databases were created from sequence repositories obtained from the corresponding database websites. Trimmed V1-2 and V3-4 reads for all strains were aligned to all 16S databases by the command-line BLAST+ toolkit 2.12.0 using BLASTn algorithm [22]. Resulting tables were sorted by sequence identities and mismatches.

Equivalently best matching species of all databases and both 16S regions were analyzed. Results were scored according to the following scheme. A total of 25 (V1-2) or 26 (V3-4) points were regarded as maximum, when all isolates could be unequivocally classified towards the species, which means the sequences of the species sought was the best match and distinguishable from closely related species. One point was deduced if a) the best matches were not distinguishable from closely related genera or families b) closely related species were not present or c) sequences were only designated towards lower taxonomic levels like the genus or family level. Final scores for all pipelines and both 16S regions were summarized to compare their performance regarding species-level identification.

## Results

We studied the species- and genus-level accuracy of 16S-based microbiome sequencing in a very simplified and defined experimental setting, allowing the precise investigation of analytical influences on species- and genus-level identification. A 100% relative abundance of the corresponding species was expected if all sequencing reads are classified correctly towards the species level, and a proportion of 95 percent was regarded as an acceptable classification result. Applying that cutoff, 24 (92%) out of 26 samples showed at least one inaccurate result, with regard to the analysis pipelines used or to the variable regions analyzed (Table 3). Across all pipelines tested, between 5 and 16 samples (20 to 64%) were correctly assigned to the species level using the V1-V2 region, but only 5 to 9 (19 to 35%) when targeting V3-V4. The pipeline using the Silva database did not yield species-level results at all. Accurate genus-level results were observed for 24 (d2.LTP), 24 (d2.GTDB), 23 (SG), 22 (d2.SILVA), and 22 (vs.GG) out of 25 isolates in the V1-2 region, and for 24 (d2.LTP), 20 (d2.GTDB), 21 (SG), 20 (d2.SILVA), and 23 (vs.GG) of 26 isolates in the V3-4 region.

**Table 3:** Correctly identified isolates on species or genus level using five different analysis pipelines.

Pipeline/Database	d2.GTDB	d2.LTP	d2.SILVA	SG	vs.GG
N species assigned **V1-2** (max 25)	13	16	0	14	5
N genus assigned **V1-2** (max 25)	24	24	22	23	22
N species assigned **V3-4** (max 26)	7	9	0	8	5
N genus assigned **V3-4** (max 26)	18	24	20	21	23
N species assigned **Total** (max 51)	**20**	**15**	**0**	**12**	**10**
N genus assigned **Total** (max 51)	**42**	**40**	**42**	**44**	**45**

**Fig 1:**
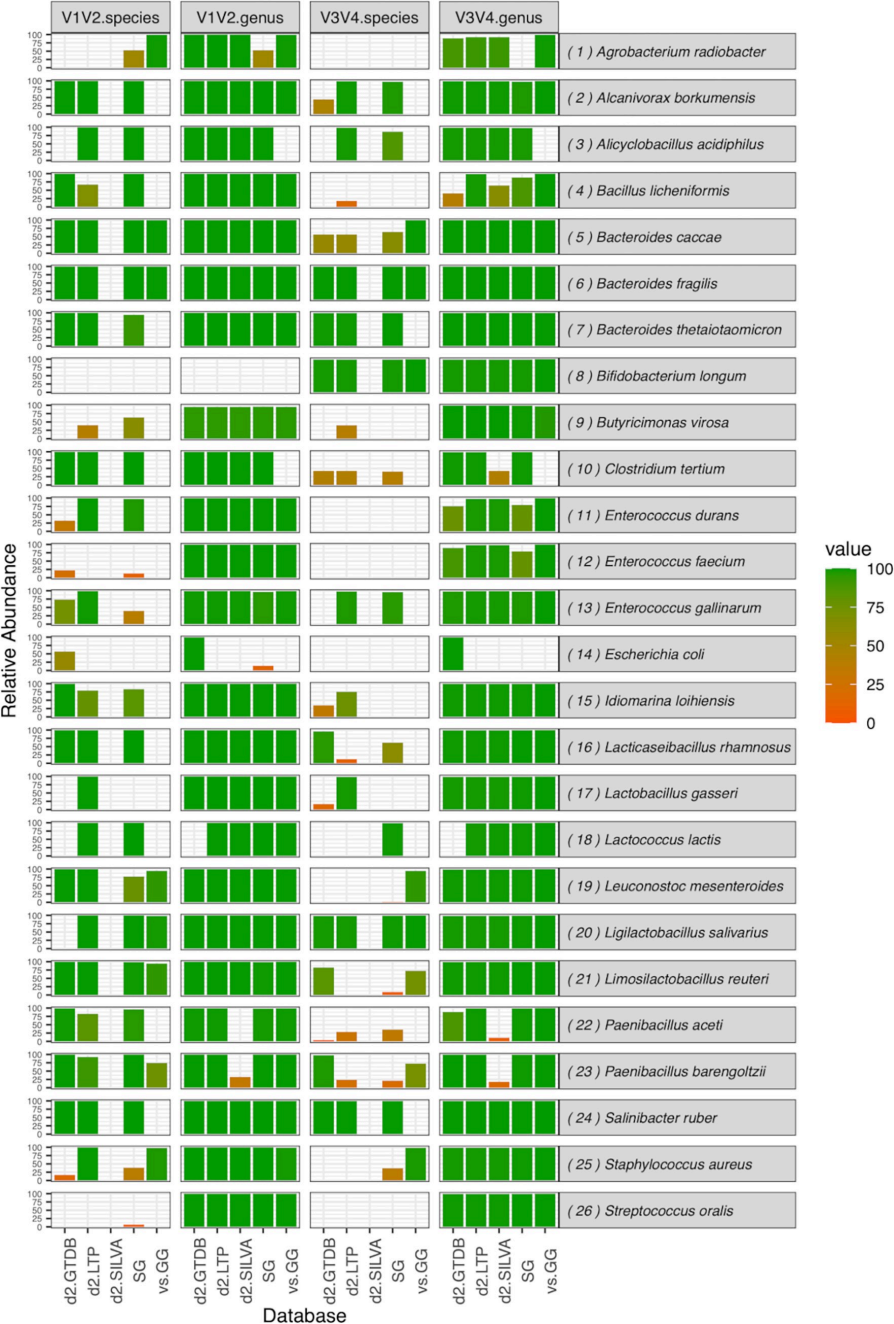
Fractions of correctly assigned sequences on the species- and genus-level after sequencing of V1-2 and V3-4 16S rRNA gene regions and sequence analysis using different pipelines and 16S rRNA reference databases. SG = SmartGene IDNS pipeline, LTP = All Species Living Tree database, GTDB = Geome Taxonomy Database, GG = Greengenes Database, SILVA = SILVA database.

The detailed results per strain are summarized in Fig 1. We selected representative cases to provide examples of factors which impact classification accuracy in S1 File.

For the sample containing *Bifidobacterium longum* (8), no PCR amplification was observed in the V1-2 region, so only the V3-4 region has been considered. *Bacteroides fragilis* (6) was the only isolate where results were fully in line with the expected 100% relative abundance. This was true across all pipelines and databases used, variable 16S regions analyzed and the taxonomic levels considered. For all other strains several deviations from the expected value were observed, which were dependent on the bacterial species analyzed.

More isolates were identifiable towards the species and genus-levels in the V1-2 region than in the V3-4 region. Across all analysis pipelines 48/100 (48%) results showed an accurate classification towards the species level in the V1-2 region compared to 30/104 (29%) when analyzing V3-4 regions. This was especially the case for isolates 4, 5, 10, 15, 16, 19, 21, 22, and 23.

In addition to the results shown in Table 3, some pipelines and databases failed to identify specific isolates (i.e. nearly 0 percent of correctly assigned reads). Genus-level identification for both 16S regions was not possible in three cases using the GG database (isolates 3,10, and 14), while the d2.GTDB pipeline failed to identify isolate 18 (*Lactococcus lactis*). Both *Paenibacillus* species (isolates 22 and 23) exhibited very low relative abundances (V1-2: 0% and 21,8%; V3-4: 10,8% and 18%, respectively) using d2.SILVA, due to assignment of *Paenibacillus* spp. to two different genera (*Fontibacillus*/*Paenibacillus*) in the SILVA database.

Some isolates could not be classified using vs.GG while other pipelines showed nearly perfect matches. Indeed *Alcanivorax borkumensis* (2), *Alicyclobacillus acidiphilus* (3), *Bacillus licheniformis* (4), *Bacteroides thetaoitaomicron* (7), *Clostridium tertrium* (10), *Idiomarina loihiensis* (15), *Lacticaseibacillus rhamnosus* (16) and *Salinibacter ruber* (24) were missed at the genus-level (3), solely at the species-level (2, 4, 7, 15, 16, 24), or at both taxonomic levels (3,10) using vs.GG. For isolate 3 neither species- nor genus-level entries were present in the database (3). For other strains, taxonomies were deposited only towards the genus-level (2, 4, 7, 10, 15, 16, 24) which hindered species-level classification.

Considering thus the different databases and their composition with respect to the deposited 16S rDNA sequences and their corresponding taxonomic annotation, some of the sequences were not accurately assigned towards the species level by the pipelines used. In order to address this issue and to validate the previously obtained species by including the underlying database structure, we analyzed the SG, GTDB, LTP and GG databases which theoretically should allow for the assignment towards the species level. We used BLASTn to align V1-V2 and V3-V4 16S rDNA regions of all bacterial isolates (1–26) to all entries in the reference databases. Equivalent best matches were analyzed, using penalty criteria described in the Methods section. For each of these criteria one point was deduced from the maximum achievable score, i.e. a perfect annotation of 26 (V3-V4) or 25 (V1-V2) isolates towards the species level. The reassessed results are summarized in Table 4. Taking the results for both 16S regions into account, the SG pipeline and the d2.LTP pipeline performed best, while d2.GTDB achieved a lower score because 16S sequences were identical with other species or genera. For example, best matching V1-2 and V3-4 sequences in the GTDB database of *Staphylococcus aureus* were 100% identical with a sequence annotated as *Pararheinheimera mesophila*. Pipeline vs.GG performed worst, mainly because of taxonomic assignments of multiple identical database entries towards the genus-level or lower taxonomic ranks. A detailed overview of all observations obtained, and scoring is available in S1 Table.

**Table 4:** Re-assessment of pipeline performances for species assignment based on achievable resolutions within the 16S rRNA gene.

Pipeline/Database	d2.GTDB	d2.LTP	d2.SILVA	SG	vs.GG
Score **V1-2** region (max 25)	16	20	NA	19	4
Score **V3-4** region (max 26)	11	17	NA	21	14
Score **Total** (max 51)	**27**	**37**	**NA**	**40**	**18**

## Discussion

In this particular exercise of determination from a single-species culture, a large number of results did not yield the expected 100% relative abundance and identification to the species level. Given these discrepancies, we investigated the systematic causes for this.

### Matching highly homologous 16S sequences and coverage of variable regions of significance

Some bacterial species such as *Staphylococcus aureus*, *S*. *schweitzeri*, *S*. *argenteus* share homologous or even identical 16S rDNA sequences [7]; such species cannot be differentiated on the basis of their 16S sequences, as one could observe with samples 12, 25 and 26 in Fig 1 (see alignments in S1 Fig).

Other species differentiate rather within one variable region of the 16S but not elsewhere: Sample 10 containing *Clostridium tertium* also matched other close *Clostridium* species in V3-4, thus decreasing the specific abundance for *C*. *tertium*. Results in general and those obtained for strains 4, 11, 22 (Fig 1) in particular indicate a better performance of the V1-2 variable region to assign correct species, whereas V3-4 yields correct result on the genus-level only. The better performance of the V1-V2 region of the 16S was also reported by other recent studies [6] and somehow contrasts with the tendency in the field of using 16S V3-4 for targeted microbiome analyses [23, 24].

In few cases, 16S sequences can be identical even across two genera, as is with *Escherichia coli (E*. *coli)* and *Shigella flexneri*, or with *Agrobacterium radiobacter*, *Agrobacterium salinitolerans* and *Rhizobium pusense*. In these cases, we recommend results to be reported at the lowest taxon level which can be unambiguously assigned, here the family. Notwithstanding its complex diversity and ambiguous taxonomic position, *E*.*coli* strains are often part of reference materials or community standards (e.g. the Gut Microbiome Whole cell Mix (American Type Culture Collection, ATCC), ZymoBIOMICS microbial community standards (Zymo Research)).

Finally, when contigs are shortened, e.g. after quality trimming, they may lack sections of sequence with discriminatory capacity. This can easily result in assignment to different taxon levels: the longer contigs may match to the species and the shorter ones to the genus-level, thus yielding a lower fraction of correctly assigned sequences to the species level, but not for the genus (see results for *Lactobacillus rhamnosus* in V3-4 of the SG pipeline with sample 16). We therefore recommend to assess the relative abundance on both levels, species and genus, especially if the presence of other close species is unlikely for the specimen. Still, guidelines for quality filtering or trimming of 16S sequencing data are still missing.

### Deficiencies in matching algorithms and relevant criteria

Matching of reads is greatly influenced by their length and accuracy. OTU based approaches cluster similar contigs towards a defined sequence identity (“binning”) before matching the consensus to the database. Provided a predefined similarity for binning at 97%, this means that 500 bp contigs with up to 15 mismatches will be clustered together, thus reducing the ability to differentiate close species when present in the sample [25, 26]. The SG pipeline of SmartGene only clusters identical contigs and matches these against its proprietary Centroid database, thus not compromising by binning the few but crucial mismatches for the differentiation of close species, as seen for strains 9 and 18. Non-clustering approaches (e.g. SmartGene) or de-noising approaches like zero-radius OTUs (zOTUs) and amplicon sequence variants (ASV) applying the unoise [19] and dada2 [15] approach, theoretically allow differentiation of species with sequences towards single nucleotide resolution.

Match consistency indicates that the closest matching species to a contig belong to the same genus and is thus indicative of a high confidence in genus matching. If the closest matching species belong to several genera, it suggests that the sequenced region is either not being discriminative enough for the type of organisms encountered or that taxonomy is uncertain within the organisms encountered. Only in the SG pipeline is the match consistency accounted for in the algorithm of assigning species and genera. An example showing inconsistency of matches is sample 1 (*Rhizobium*) or sample 14 (*E*. *coli*).

### Database design and curation, species-coverage

Several factors determine the usefulness of a reference database for microbiome 16S contig matching. First, annotation accuracy assures that the correct species names are attached to the sequences, in compliance with up-to-date bacterial nomenclature. Sample 16, containing *Lacticaseibacillus rhamnosus*, may serve as an example for divergent results: pipelines d2.SILVA and vs.GG identified the sample under its former name *Lactobacillus rhamnosus* and therefore does not yield the correct genus.

Second, the content should be fully representative of the Kingdom, to ensure that all relevant species are included in a database to cover the natural diversity of the bacterial kingdom adequately. Missing a species in the database can result in either misclassification by matching the contigs to the next closest species instead, or in an identification to a higher taxonomic rank. The *S*. *aureus* (strain 25 in Fig 1) in our study illustrates an apparently accurate assignment of reads in LTP and GG for V1-2; however, since no sequences for *S*. *argenteus or S*. *schweitzeri* were present in these databases, this match is not exhaustive and thus not reliable. The correct result should be *Staphylococcus “*genus”, given that *aureus*, *argenteus and schweitzeri c*annot be reliably differentiated within the variable regions analyzed. The same is true for *E*. *coli*, where sequences of *Shigella dysenteriae*, *E*. *fergusonii* etc. were missing in the databases; the correct result would have to be *Enterobacteriacae* family, given the equivalent matches to different genera.

Some authors advocate for databases focusing on pathogenic or clinically relevant species, or on specific habitats: examples are the human intestinal 16S rRNA database (HITdb, [27], or the expanded Human Oral Microbiome Database (HOMD, [28]). These databases often miss naturally occurring variants and other close species and thus will likely not detect contaminants or unusual infections. That intra-species diversity has an impact on match accuracy of contigs and thus should also be covered by a database for easier species differentiation [7]. A type strain database such as LTP does not sufficiently cover this diversity, as illustrated by the reduced relative abundance of *Bacillus licheniformis* (strain 4) in the V1-2 region. Some pipelines rely on clustered reference databases, such as Greengenes [29], which cluster entries at a certain sequence identity (e.g. 97%) to reduce computing time.

Curating a database, for updates and for adoption of current nomenclature, is often manually performed by experts. This mode of curation is rather flexible to adopt new taxa and modified nomenclature, but is very demanding in time and expertise, which will not allow for covering all taxa equally. Furthermore, expert curation requires perpetual funding, which is not guaranteed via research grants. Commercial solutions on the other hand often lack the transparency on their criteria for including entries and on the frequency and thoroughness of updates. For all manually curated databases, a certain lag behind most recently published species and nomenclature can be expected. The Greengenes database is outdated (latest release date is 2013) and its use is discouraged. However, it is still used occasionally. Algorithm-curated databases such as SmartGene’s 16S Centroid databases hold the promise of more frequent updates with adequate representation of natural diversity. In order to appreciate the quality of the curation, users should have access to its principals, to a list of species covered in the database, the number of sequences evaluated to select representative records and be aware of the frequency of updates.

Nomenclatural changes can alter measurements of relative abundances, as was observed with sample 1, containing *Agrobacterium radiobacter* (formerly *Rhizobium radiobacter*) with the last update of the most up-to-date SG pipeline: since some of the shorter contigs also matched the next closest species of the genus *Rhizobium*, this reduced the overall fraction of sequences assigned correctly to the genus *Agrobacterium*.

### Analytical process and sequencing technologies

The design of appropriate PCR primers is crucial for the recovery of species and can lead to missing an organism or induce an amplification bias which subsequently leads to shifts of microbial compositions and eventually to divergent and unexpected results [5]. The same is true for DNA extraction processes, which can fail to lyse some bacterial cells or favor the recovery of certain genomes over others [30]. When relying on sequencing technologies which produce rather short reads, contigs will only cover one or two variable regions of the 16S, thus reducing the ability to differentiate a number of bacterial species; in such situation it is of importance to choose the covered regions carefully to differentiate the expected species unambiguously. As explained above, longer contigs may be assigned to the correct species whereas the shorter ones may match two and more species, reducing the calculated relative abundance for the expected species. In our study, several examples illustrate this behavior, e.g. with regard to the SG pipeline: 1, 9, 13, 19. Long read sequencing at high accuracy will certainly improve the species resolution provided that these are adequatically represented in the database used. One interesting approach was published by Karst et al., who have developed and applied a synthetic long read protocol for sequencing of full-length 16S rRNA genes [31]. However, we think that some principal problems will still remain for analyses which rely on comparison with reference databases as such databases do not fully cover the full biological diversity. The carefull selection and analysis of the reference databases related to the representativity for the species of interest will remain a very important task.

Base-calling errors impact on the matching accuracy as well. Here we used the IDTAXA (LTP, SILVA, GTDB) and SINTAX (GG) approach which are based on machine-learning algorithms using the reference databases as training sets to calculate confidence values, based on repeated random sampling of *k*-mers belonging to the query sequence. However, the training of the classifier is very much dependent on the composition and reliability of the reference database used.

Ion Torrent^®^ semiconductor-based sequencing was used in this study, while the Illumina MiSeq^®^ platform is certainly the most widely used platform for 16S rDNA gene sequencing. Our data demonstrates, that the observed differences can be well explained on the basis of DNA sequence alignments or the underlying database structure and the findings of our study should therefore be also applicable to other short read technologies. Strains that are well distinguishable on the basis of 16S rRNA genes could be well identifiable at the species level, indicating a high accuracy of the chosen method. Although the Ion Torrent^®^ platform might produce higher rates of indel errors, especially in homopolymer regions [32], comparable performance for 16S amplicon sequencing to Illumina-based workflows has been demonstrated [33]. However, quality filtering steps and subsequent bioinformatic methods should be adapted to the technology used and validated.

### The choice of strains for quality control

In our study we were relying on strains from strain collections with the exception of one strain of Butyricimonas virosa (9), which originated from a patient specimen (blood culture) and was unambiguously identified by MALDI. Microbiome results obtained with the SG and the LTP pipelines however indicated presence of a minority of several Butyricimonas species, e.g. B. virosa (63.7%), faecihominis (21.25%) and paravirosa (9.45%), and of a taxonomically rather different bacterium, Catabacter hongkongensis (4%), which was unambiguously identified by its signature sequence in the V1-2 region (see alignment in supplemental materials). When inquiring about the case retrospectively, the patient history revealed a sepsis after surgical intervention on recurrent colon diverticulitis, with peritonitis as complication after perforation. In light of such clinical picture, there is indeed a strong possibility of disseminating gut flora via the blood stream; the microbiome results were rather plausible for the case and may have helped clinicians in their management of the case. Note that only the SmartGene and LTP pipelines were able to detect the mixed infection, whereas the other pipelines did not. The V1-2 primers were not able to amplify the Bificobacterium longum (9) DNA. However, primer mismatches in the 27f primer region to some Bifidobacterium species have been reported before [34]. Sequence alignments of full-length 16S rRNA gene sequences of the most frequently occurring Bifidobacterium species indicate, that the V1-2 and the V3-4 regions are both discriminative for the differentiation of such closely related species. In general, primers should be validated in silico and in vitro to verify the coverage and amplification of relevant species for the environment being tested.

## Recommendations

To conclude our analysis, we recommend the following:

- The target regions for sequencing should enable sufficient differentiation of all species which are expected to be detected, especially if the sequencing technology used produces short contigs. The V1-2 region of the 16S rRNA gene performs generally better for species identification than the V3-4 region.
- Assessing read quality and subsequent filtering/trimming should be transparent, in order to enable better understanding and troubleshooting of the results obtained.
- Reference databases used for analyses of microbiomes should be representative of all bacterial species and their natural diversity and include potential contaminants; annotations should follow up-to-date nomenclature. We suggest to avoid the use of targeted and thus restricted databases and opt for covering maximal diversity to allow for accurate and significant matches.
- Analysis pipelines and databases used should be versioned and validated. Any changes in pipelines or databases including updates should be subject to new validation using a suitable validation dataset which includes reads from real sequencing runs.
- Matching contig against a reference database should yield all equivalent matches, up to the next closest species which can be ruled out given the presence of significant mismatches. All such close matches should be listed as alternative results.
- Contigs which cannot be assigned unambiguously to any species should be assigned to list of all species that can be considered or to a genus or family, depending on the certainty of the matches obtained at such higher taxonomic levels.
- Quantitation of abundance should include assessment on both, the species and the genus-level; attention should be paid to shorter contigs which may only match at the genus-level. Quantification of abundance should be verified for inconsistencies especially if closely related taxa are in the same specimen and match ambiguously.
- Strains to be used for microbiome standardization or performance assessment should be fully characterized, to identify the species beyond doubt and to rule out contamination. One should take into account the ability to differentiate other close species and genera and avoid organisms of unclear taxonomy; expected results should also account for species with pronounced intra-species or intra-operon diversity.

## Supporting information

S1 Fig Sequence alignments of 16S rRNA genes for selected bacterial strains. (PDF)

S1 File Results for data analysis for additional bacterial isolates. (DOCX)

S1 Table Re-assessment of pipeline performances for species assignment based on achievable resolutions within the 16S rRNA gene. (DOCX)
