# Supplementary material for: Reliability of species detection in 16S microbiome analysis: Comparison of five widely used pipelines and recommendations for a more standardized approach
Source: PLoS One. 2023 Feb 16;18(2):e0280870. doi: 10.1371/journal.pone.0280870 (PMC9934417; doi:10.1371/journal.pone.0280870)
Supplement: S1 Fig — (PDF) [file pone.0280870.s001.pdf]

]

Agrobacterium radiobacter (1)

]

Bacillus licheniformis (4)

]

Butyricimonas virosa (9)

]

Clostridium tertium (10)

]

F Enterococcus durans/faecium/gallinarum (11,12,13)

]

Escherichia coli (14)

]

Lacticaseibacillus rhamnosus (16)

]

Staphylococcus aureus (25)

]

Streptococcus oralis (26)
