# Supplementary material for: Reliability of species detection in 16S microbiome analysis: Comparison of five widely used pipelines and recommendations for a more standardized approach
Source: PLoS One. 2023 Feb 16;18(2):e0280870. doi: 10.1371/journal.pone.0280870 (PMC9934417; doi:10.1371/journal.pone.0280870)
Supplement: S1 File — (DOCX) [file pone.0280870.s002.docx]

**S2 File Supplementary Results**

Sequencing of ***Bacillus licheniformis (4)*** showed high relative abundance on the species-level only when analyzing the V1-2 region, but not for the V3-4 region. Except for the vs.GG pipeline (0%), the other pipelines were effective in identifying *B. licheniformis* in the V1-2 region (SG: 99.6, d2.LTP:67.2 and d2.GTDB:99.8 percent relative abundance). A total of 12.2 percent of total reads were classified to “unclassified Bacillus” using the d2.LTP pipeline. In the V3-4 region, only the d2.LTP pipeline showed >0 percent relative abundance (18.2 %). For the V3-4 region, also genus-level abundances were significantly lower compared to analyzing V1-2 reads. Sequence alignment of full-length 16S sequences using centroid sequences within the SG IDNS module revealed nearly identical sequences for *Bacillus spp.* in the V3-4 region. *Bacillus licheniformis* and *Bacillus swezeyi* share identical sequences in V1 but differentiate with several mismatches in V2, thus allowing reliable species abundance provided that the reads cover both regions. In V3-4, however, there is a very high homology between several *Bacillus* species.

The sample containing ***Clostridium tertium (10)*** showed a better performance for V1-2 than V3-4 region. Nearly 100 % of V1-2 reads could be classified towards the species level using the SG, d2.LTP and d2.GTDB pipeline, while V3-4 revealed 40.7, 42.5 and 42.5 percent respectively. Neither species nor genus-level detection was possible at both regions using the vs.GG database. Analysis of sequence alignments of full-length 16S centroid sequences demonstrated that differentiation of *Clostridium tertium* can be achieved in the V1-2 region but not in the V3-4 region, due to high homology with e.g. *C. chauvoei* and *C. septicum* and others,, which yields only genus matches reliably. Screening the GG database revealed the absence of *Clostridium* species leading to 100% abundances only on the family level.

***Paenibacillus*** ***aceti* (22)** and ***barengoltzii* (23)** were accurately quantified using V1-2 but not by the V3-4 region. In the V1-2 region, only the GG database failed to detect *P. aceti* at the species-level. In the V3-4 region all pipelines showed below 50 % abundances except for d2.GTDB; which enabled more reads to be correctly classified (97.7 %) for isolate 23. At the genus-level, significantly lower abundances were obtained for both species using d2.SILVA (0 % and 32.8% on the V1-2 and 10.8 % and 8 % on the V3-4 region respectively). These reads were mis-classified as *Fontibacillus* species. *Paenibacillus aceti* and *Paenibacillus barengoltzii* share dissimilarity in the 16S V1-2 region to other *Paenibacilli* However, the v3-4 region shows significant homology, including other species, such as *P. vini.*

The sample containing ***Agrobacterium radiobacter (1)*** showed discrepancies in the genus-level quantification using the SG pipeline at both variable regions (V1-2: 53 % and V3-4: 0.03 %) while all other databases resulted in classification of nearly 100 % of total sequencing reads. (99.2 %). As alignment of full-length 16S sequences revealed *(See Suppl. Figure 1)* in V1-2, the sequence homology is high with regard to *Rhizobium pusense* and *Agrobacterium salinitolerans*. Reads match several species and genera and thus quantification on species and genus-level is not possible; family abundance yields 99.9%. For the v3-4 region, even less diversity is observed between *Agrobacterium spp* and *Rhizobium spp*. On the species level, correct identification was only possible in the V1-2 region using the GG pipeline, while all other pipelines resulted in nearly 0 percent relative abundance, except SG pipeline, which showed 53% abundance in the V1-2 region.

Nearly 100 % of the reads were classified towards the genus-level for the ***Butyricimonas virosa*** *(9)* sample. However species-level identification was almost impossible in both regions. This species could not be identified on both regions using the d2.GTDB and vs.GG pipeline, while SG and d2.LTP showed relative abundances of 63.7 and 0.03 on the V1-2 region and 40.5 and 40.1 on the V3-4 region respectively. Further analyzing the sequencing reads in the SG pipeline revealed, that this sample contained a mixture of several *Butyricimonas* species (*virosa* (63.7%), *faecihominis* (21.25%) and *paravirosa* (9.45%)), along with *Catabacter hongkongensis* (4%) in the V1-2 region, where these species are easily differentiated by the 16S rRNA gene diversity. *Catabacter* species could be identified in similar abundances in V1-2 sequencing reads using the SG, d2.SILVA, d2.LTP and GTDB pipeline while reads were classified to *Christensenella* species using vs.GG. Looking at V3-4 region, only the SG pipeline was able to identify this species at 0.7 % relative abundance.

Three samples contained different ***Enterococcus*** species: *E. durans (11), E. faecium (12) and E. gallinarum (13).* All three species were accurately quantified at the genus level only. All databases showed nearly 100% genus-level quantities which was slightly lower for isolates 11 and 12 at the V3-4 region using the SG (80% and 79.4 %) and d2.GTDB (90 % and 97.8 %) pipeline. Species-level classification of 11 and 12 *w*as much less precise on the v3-4 region. In this region, species level identification failed for all databases used, while for isolate 13 the SG and d2.LTP pipeline showed nearly 100% abundances. Sequence alignment revealed, that species 11 and 12 share identical nucleotide sequences in the V3-4 region and in addition cannot be discriminated from *E. hirae and E. ratti*. In the V1-2 region, *E. faecium* and *E. durans* were dissimilar only in 2 nucleotide positions, while *E. gallinarum* can be discriminated in the v2 region at multiple positions. However *E. gallinarum* and *E. casselivlavus* can be discriminated by only one single nucleotide position (A-T mismatch at position 297) in the V1-2 region.

The sample containing ***Staphylococcus aureus* (25)** showed perfect identification on the genus-level. More than 95 percent of all reads were correctly classified towards the species level only for the GG database (V1-2 and V3-4) region and the d2.LTP pipeline (V1-2 region only). Using other databases revealed 38.6 and 36.5 (SG), or 16.7 and 0 percent (d2.GTDB) in the V1-2 and V3-4 region, respectively. The d2.LTP pipeline allowed classification of no reads (0 % relative abundance) in the V3-4 region. *S. aureus* 16S rRNA gene sequences are almost identical with *S. argenteus* and *S. schweitzeri* in the V2 region; no differences are observed in the V3 and V4 regions. The group of these three species is however rather distinct from other *Staphylococcus spp* (see alignment, Suppl Figure 1). Most reads (62%) were associated with all three species in V1-2 region, thus yielding a genus result, a minority (38%) of reads mapped within the known diversity of *S. aureus,* which is not observed for *S. argenteus* and *S. schweitzeri*. In addition, analysis of the vs.GG database showed that sequences of closely related species S*.schweitzeri* and *S.argenteus* are missing, and sequences classified as *S. aureus* were over-represented (50,501 out of 1,012,863 sequences).

The sample containing ***Streptococcus oralis* (26)** could only be classified on the genus level. Here, nearly all reads could be correctly classified using five different databases. On the species level, no match (0 % relative abundance) was observed for most databases on both regions. Only the SG pipeline allowed classification of 6.8 % of total reads in the V1-2 region. Alignment of Centroid sequences with the SmartGene IDNS module showed that *Streptococcus mitis* and *oralis* share identical nucleotide sequences in the v3-4 region and are dissmiliar only in one position in the V1-2 region (position 273). Other close species such as *S. uberis* or *S. cristatus* can be differentiated because of mismatches in the v2 region.

V1-2 as well as V3-4 reads obtained for the sample containing a ***Lactococcus lactis* (18)** isolate were misclassified as *Brachyspira hampsonii* when using the dada2 pipeline together with the GTDB reference database (99.7 and 98.7 % respectively). Comparisons of full-length 16S rDNA sequences extracted from the GTDB database revealed that one database entry was incorrectly annotated as *Brachyspira hampsonii* B (GTDB Identifier GCF_001746205.1 which was obtained from Genbank genome assembly GCA_001746205.1) and shared 100 percent sequence identity with *Lactococcus lactis* using the SmartGene IDNS module. However *L. lactis* was correctly identified using the recently released r202 version of the GTDB (data not shown).

For ***Ligilactobacillus salivarius* (20)**, no species-level classification was possible for the V1-2 region when using d2.GTDB. Here nearly all reads were misidentified as *Ligilactobacillus hayakitensis* although several sequences assigned to *L. salivarius* were present in the database.

Reads from the sample containing an ***Escherichia coli*** strain **(14)** could not be classified towards the species or genus level but only towards the family level in both variable regions. Only with d2.GTDB, nearly all reads were classified towards *Escherichia* genus and 57.1 to the species *Escherichia coli* in the V1-2 region. The SG pipeline identified 14.5 % of total reads to the genus-level in the V1-2 region. Sequence alignment of centroid sequences revealed, that *Escherichia coli*, *Escherichia fergusonii* and several *Shigella spp*. (incl *boydii, dysenteriae, flexneri*) share identical or almost identical 16S rRNA gene sequences. Analyzing the taxonomic distribution of *Escherichia* and *Shigella*-matching sequences showed, that the GTDB database contained no *Shigella*, but only *Escherichia* species. All *Shigella* species were designated as *Escherichia* genus (e.g. *Escherichia flexneri, Escherichia dysenteriae,* ...).
