# Supplementary material for: Reliability of species detection in 16S microbiome analysis: Comparison of five widely used pipelines and recommendations for a more standardized approach
Source: PLoS One. 2023 Feb 16;18(2):e0280870. doi: 10.1371/journal.pone.0280870 (PMC9934417; doi:10.1371/journal.pone.0280870)
Supplement: S1 Table — (DOCX) [file pone.0280870.s003.docx]

**S3 Table:** Re-assessment of pipeline performances for species assignment based on achievable resolutions within the 16S rRNA gene

| V1-2 | | | | | | |
| --- | --- | --- | --- | --- | --- | --- |
|  |  | d2.GTDB | d2.LTP | SG | vs.GG | Comments V1-2 |
| 1 | *Agrobacterium radiobacter* | 1 | 0 | 0 | 1 | GTDB: A. rhizogenes identical, GG: only genus-level available |
| 2 | *Alcanivorax borkumensis* | 0 | 0 | 0 | 1 | GG: species not in db |
| 3 | *Alicyclobacillus acidiphilus* | 1 | 0 | 0 | 1 | GTDB: species not in db, GG: species should be correctly identified |
| 4 | *Bacillus licheniformis* | 0 | 1 | 0 | 1 | LTP:identical seq Bacillus aerius, GG: seq available, but only genus level assignment |
| 5 | *Bacteroides caccae* | 0 | 0 | 0 | 0 |  |
| 6 | *Bacteroides fragilis* | 1 | 0 | 0 | 0 | GTDB: identical sequence to Bacteroides bouchesdurhonensis |
| 7 | *Bacteroides thetaiotaomicron* | 0 | 0 | 0 | 1 | GG: only genus level available |
| 8 | *Bifidobacterium longum* | NA | NA | NA | NA |  |
| 9 | *Butyricimonas virosa* | 1 | 0 | 0 | 1 | GTDB: identical sequence to Butyricimonas faecihominis, GG: only genus-level available |
| 10 | *Clostridium tertium* | 0 | 0 | 0 | 1 | GG: identical seq with family level assignment |
| 11 | *Enterococcus durans* | 0 | 0 | 1 | 1 | GTDB: Enterococcus_B faecium_B best match, SG: lactis and durans identical seqs,  GG: only genus-level available |
| 12 | *Enterococcus faecium* | 0 | 0 | 1 | 1 | SG: 1 mismatch to Enterococcus hirae, GG: only genus-level available |
| 13 | *Enterococcus gallinarum* | 0 | 0 | 1 | 1 | SG: 3 mm to Enterococcus casseliflavus, GG: only genus-level available |
| 14 | *Escherichia coli* | 1 | 1 | 0 | 0 | LTP: Shigella spp. Best match, E.coli with mismatches,  GTDB: All shigella spp are named escherichia spp |
| 15 | *Idiomarina loihiensis* | 1 | 0 | 1 | 1 | GTDB: Idiomarina abyssalis best match, SG: Idiomarina ramblicola with 0 mm |
| 16 | *Lactobacillus rhamnosus* | 0 | 0 | 0 | 1 | GG: only g__Lactobacillus s__zeae in database |
| 17 | *Lactobacillus gasseri* | 0 | 1 | 0 | 1 | LTP: L. Paragasseri best match, GG: only genus-level available |
| 18 | *Lactococcus lactis* | 1 | 0 | 0 | 1 | GG: only genus-level available |
| 19 | *Leuconostoc mesenteroides* | 1 | 0 | 1 | 1 | GTDB: Leuconostoc fallax identical, SG: correct, but below 95 % cutoff, GG: genus with 0 mismatches |
| 20 | *Lactobacillus salivarius* | 1 | 0 | 0 | 1 | GTDB: speies not present. Best match Ligilactobacillus hayakitensis, GG: genus with 0 mismatches |
| 21 | *Lactobacillus reuteri* | 0 | 0 | 0 | 1 | GG: genus with 0 mm |
| 22 | *Paenibacillus aceti* | 0 | 1 | 0 | 1 | LTP: below 95 % cutoff, GG: only genus-level available |
| 23 | *Paenibacillus barengoltzii* | 0 | 0 | 0 | 1 | GG: correct, but below 95 % cutoff |
| 24 | *Salinibacter ruber* | 0 | 0 | 0 | 1 | GG: only genus-level available |
| 25 | *Staphylococcus aureus* | 1 | 0 | 0 | 1 | GTDB: Pararheinheimera mesophila best match, GG: genus with 0 mismatches |
| 26 | *Streptococcus oralis* | 0 | 1 | 1 | 1 | LTP: S. oralis not best match,SG: 1 mm to S. Mitis, GG: genus with 0 mm |
| **Sum V1-2 Penalties** | | **9** | **5** | **6** | **21** |  |
|  |  |  |  |  |  |  |
| V3-4 | | | | | | |
| Strain | Species | d2.GTDB | d2.LTP | SG | vs.GG | Comments V3-4 |
| 1 | *Agrobacterium radiobacter* | 0 | 0 | 0 | 0 | SG: A. Arsenijevicii and R. nepotum identical, LTP: A. nepotum, fabacearum and  arsenijevicii identical, GTDB: several Agrobacterium species identical, GG: only genus-level available |
| 2 | *Alcanivorax borkumensis* | 1 | 0 | 0 | 1 | GDDB: 1mm to Alcanivorax sp000155615 |
| 3 | *Alicyclobacillus acidiphilus* | 1 | 0 | 0 | 1 | GTDB species not in database |
| 4 | *Bacillus licheniformis* | 0 | 0 | 0 | 0 | GG: only genus-level available, one staphylococcus seq with 0 mm |
| 5 | *Bacteroides caccae* | 1 | 1 | 1 | 0 | Differentiation possible for all DB, GTDB and SG below 95 % cutoff |
| 6 | *Bacteroides fragilis* | 1 | 0 | 0 | 0 | GTDB: Schaedlerella sp004556565 identical |
| 7 | *Bacteroides thetaiotaomicron* | 0 | 0 | 0 | 1 | GG: only genus-level available |
| 8 | *Bifidobacterium longum* | 0 | 0 | 0 | 0 | SG: B.breve centroide identical but shorter seq. (290bp) |
| 9 | *Butyricimonas virosa* | 1 | 0 | 0 | 0 | GTDB: best match 11mm, no butyricimonas |
| 10 | *Clostridium tertium* | 1 | 1 | 1 | 1 | Differentiation possible for all databases, GTDB, SG and GG below 95 % cutoff |
| 11 | *Enterococcus durans* | 1 | 0 | 0 | 1 | GG:only genus-level available |
| 12 | *Enterococcus faecium* | 1 | 0 | 0 | 0 | GTDB: Sphingomonas sp003946805 identical sequence |
| 13 | *Enterococcus gallinarum* | 1 | 0 | 0 | 0 | GTDB: Enterococcus_D sp002140915 best match |
| 14 | *Escherichia coli* | 1 | 0 | 0 | 0 | GTDB: Tumebacillus flagellatus with 0mm (additional genus) |
| 15 | *Idiomarina loihiensis* | 0 | 1 | 0 | 0 | LTP: should be differentiable from Idiomarina abyssalis |
| 16 | *Lactobacillus rhamnosus* | 0 | 1 | 1 | 1 | SG: correct, but below 95 % cutoff |
| 17 | *Lactobacillus gasseri* | 0 | 1 | 0 | 1 | LTP: L. paragasseri and hominis identical |
| 18 | *Lactococcus lactis* | 1 | 1 | 0 | 0 | GTDB: Brachyspira hampsonii_B with 0 mismatches |
| 19 | *Leuconostoc mesenteroides* | 0 | 0 | 0 | 1 | GG: genus with 0 mm |
| 20 | *Lactobacillus salivarius* | 0 | 0 | 0 | 1 | GG: genus with 0 mm |
| 21 | *Lactobacillus reuteri* | 1 | 1 | 0 | 1 | ltp: 2nd match with 2mm |
| 22 | *Paenibacillus aceti* | 1 | 1 | 1 | 0 | GG: only genus-level available |
| 23 | *Paenibacillus barengoltzii* | 1 | 1 | 1 | 0 | Differentiation possible for all DB but GG, GTDB and SG below 95 % cutoff |
| 24 | *Salinibacter ruber* | 0 | 0 | 0 | 1 | GG: only genus-level available |
| 25 | *Staphylococcus aureus* | 1 | 0 | 0 | 1 | GTDB: Pararheinheimera mesophila best match |
| 26 | *Streptococcus oralis* | 0 | 0 | 0 | 0 | GG: only genus-level available |
| **Sum V3-4 Penalties** | | **15** | **9** | **5** | **12** |  |
|  |  |  |  |  |  |  |
| **Total Sum Penalties (V1-2 & V3-4)** | | 24 | 14 | 11 | 33 |  |
| **Score (Max 51 Species Assigned)** | | 27 | 37 | 40 | 18 |  |
